# Intraoperative radiation therapy for early stage breast cancer

**DOI:** 10.1186/s12893-021-01427-5

**Published:** 2022-01-26

**Authors:** Vahid Zangouri, Hamid Nasrollahi, Ali Taheri, Majid Akrami, Peyman Arasteh, Seyed Hassan Hamedi, Masoumeh Ghoddusi Johari, Nazanin Karimaghaee, Aliye Ranjbar, Mohammad Yasin Karami, Sedigheh Tahmasebi, Ahmad Mosalaei, Abdolrasoul Talei

**Affiliations:** 1 Surgical Oncology Division, General Surgery Department, Shiraz University of Medical Sciences, Shiraz, Iran; 2 Breast Diseases Research Center, Shiraz University of Medical Sciences, Shiraz, Iran; 3 Radiation Oncology Department, Shiraz University of Medical Sciences, Shiraz, Iran; 4 Trauma Research Center, Rajaei Hospital, Shiraz University of Medical Sciences, Shiraz, Iran; 5 Core Medical Trainee, Northumbria Healthcare NHS Foundation /Trust, Newcastle Upon Tyne, UK; 6 Student Research Committee, Shiraz University of Medical Sciences, Shiraz, Iran; 7 Shiraz Institute for Cancer Research, Shiraz University of Medical Sciences, Shiraz, Iran

**Keywords:** Intraoperative radiation therapy, Breast cancer, Middle East recurrence

## Abstract

**Background and objective:**

We report our experiences with Intraoperative radiation therapy (IORT) among breast cancer (BC) patients in our region.

**Methods:**

All patients who received radical IORT from April 2014 on to March 2020 were included in the study. Patient selection criteria included: Age equal or older than 45 years old; All cases of invasive carcinomas (in cases of lobular carcinomas only with MRI and confirmation); Patients who were 45–50 years old with a tumor size of 0–2 cm, 50–55 years old with a tumor size of < 2.5 cm, and those who were ≥ 55 years old with a tumor size of < 3 cm; Invasive tumors only with a negative margin; Negative nodal status (exception in patients with micrometastasis); A positive estrogen receptor status. Primary endpoints included death and recurrence which were assessed using the Kaplan–Meier method.

**Results:**

Overall, 252 patients entered the study. Mean (SD) age of patients was 56.43 ± 7.79 years. In total, 32.9% of patients had a family history of BC. Mean (SD) tumor size was 1.56 ± 0.55 cm. Mean (IQR) follow-up of patients was 36.3 ± 18.7 months. Overall, 8 patients (3.1%) experienced recurrence in follow-up visits (disease-free-survival of 96.1%), among which four (1.5%) were local recurrence, two (0.8%) were regional recurrence and two patients (0.8%) had metastasis. Median (IQR) time to recurrence was 46 (22, 53.7) months among the eight patient who had recurrence. Overall, one patient died due to metastasis in our series. Eleven patients (4.3%) with DCIS in our study received IORT. All these patients had free margins in histopathology examination and none experienced recurrence.

**Conclusion:**

Inhere we reported our experience with the use of IORT in a region where facilities for IORT are limited using our modified criteria for patient selection.

**Supplementary Information:**

The online version contains supplementary material available at 10.1186/s12893-021-01427-5.

## Introduction

Intraoperative radiation therapy (IORT) has been considered as an appropriate substitute for whole breast irradiation (WBI) among patient with early stage breast cancer (BC) that have undergone breast conserving surgery (BCS) [1]. IORT includes delivering a single dose of radiation during BCS [2].

Two of the largest and well-known clinical trials include the TARGIT [3] and ELIOT studies [4]. These two clinical trials, despite having differences in treatment specifics, primarily compared IORT to WBI and evaluated the efficacy of IORT among patients with early stage BC. Different study designs and more importantly different criteria for IORT have made reports from different institutions and regions of the world variable. Furthermore, following these reports a large debate has been ongoing on the efficacy of IORT in the settings of early BC.

BCs have different genetic characteristics, clinicopathology and behavior in different geographic regions [5] and to date limited reports have been published on IORT from the Middle East [6]. In this study we aimed to report our experiences with IORT among patients with BC using data from the largest BC registry in Iran.

## Methods

### Study settings

This study was conducted as part of the Shiraz Breast Cancer Registry which is a surgical registry affiliated to Shiraz University of Medical Sciences in Motahhari Clinic, Shiraz, Iran. This is the main referral center for patient with BC in Southern and Central Iran. The registry includes data on baseline and clinical characteristics, histopathology and imaging among patients with BC. Protocol of the center has been described elsewhere [5].

### Patients

This is a retrospective report and including data from all patients who received radical IORT in our center from April 2014 on to March 2020.

In our center, IORT was first performed in April 2014. This was the second center in Iran to perform IORT and some authors of this report are members of the International Society of intraoperative radiation therapy for IORT in 2018 [7].

Our institutional guidelines were prepared with the consensus of a joint committee of experts in surgical oncology, pathology and radiation oncology and considering the guidelines of the American Society for Radiation Oncology and the American Society of Breast Surgery, ELIOT and TARGIT trials [3, 4, 8–11]. Our criteria for IORT included the following: (1) Age equal or older than 45 years old; (2) Regarding histology, all cases of invasive carcinomas were considered candidates, moreover in lobular carcinomas caution was taken and these patients were only considered candidates after MRI and confirmation of the radio-oncologist, and in cases with ductal carcinoma in-situ (DCIS) only patients with low and intermediate grade, tumor size of equal or less than 2.5 cm and a margin of 2–3 mm were considered candidates; (3) Regarding tumor size, patients between 45 and 50 years old with a tumor size of 0–2 cm, those between 50 and 55 years old with a tumor size of < 2.5 cm, and those who were 55 years old and older with a tumor size of < 3 cm were considered candidates; (4) Regarding marginal status, among those with invasive tumors a negative margin was considered sufficient and in cases of DCIS a margin of 3 mm was considered a candidate; (5) Regarding nodal status, patients with a negative nodal status (exception in patients with micrometastasis); (6) Regarding hormone receptor status, patients with a positive estrogen receptor status were considered candidates for IORT.

For the current report those with boost IORT, were excluded.

### IORT

IORT was done in our center using the Liac Sordina mobile linear accelerator. Electrons with 6, 8, 10 and 12 MeV were used according to the depth of the tumor which was measured using a marked needle. A dose of 21 Gy was administered to 95% isodose. Electron energy was chosen according to the thickness of the tissue that was prepared for radiation. Diameter of collimators was 4–6 cm and was chosen according to the diameter of the tumor and the tissue that is prepared by the surgeon for IORT [12].

### Variables and endpoints

Data on baseline characteristics (age, sex and BMI), obstetric and gynecological indices, use of oral contraceptive medication (OCP) or hormone replacement therapies, underlying diseases, social history including cigarette and alcohol use, clinical and surgery related information including type of axillary management, tumor size and grade, invasion status, estrogen (ER), progesterone (PR) and human epidermal growth factor 2 (HER2) receptor status, chemotherapy before and after surgery, histopathology information and prognosis of patients were gathered.

Endpoints were considered death and recurrence during follow-up. Local recurrence was defined as recurrence of tumor within the ipsilateral chest wall and regional recurrence was considered as recurrence within the ipsilateral axillary, infra- and supraclavicular area or within the oriental mammary lymph nodes [13].

### Follow-up

Following surgery, for the first 3 months, follow-up visits are as followed: 1 week, 1 month and 3 months after surgery. After the first 3 months and during the first 2 years after surgery, visitations are scheduled every 4 months. This is changed to every 6 months during the 2nd–5th year follow-up period. After which follow-ups are done annually.

### Ethics consideration

The study protocol was approved by the Institutional Review Board of Shiraz University of Medical Sciences. All patients gave their written and informed consent to enter the study. All study protocols followed guideline stated in the Declaration of Helsinki.

### Statistical analysis

Data were analyzed using the statistical Package for Social Sciences (SPSS Inc., Chicago, Illinois, USA), for windows, version 20. Data are reported as frequency and percentage for qualitative data and as means and standard deviations (SD) for quantitative data with normal distribution and median and interquartile range (IQR) for quantitative data without a normal distribution. The Kaplan–Meier method was used to create survival plots for recurrence (as the primary outcome).

## Results

In total, 252 patients had radical IORT during the study period in our centers and entered the study. All our patients were females. Mean (SD) age of patients was 56.4 ± 7.7 years old. In total, 32.9% of patients had a family history of BC. Mean age of pregnancy was 21.7 ± 5.4 years old.

Other baseline characteristics of patients are shown in Table 1.

**Table 1:** Baseline characteristics of patient who received IORT*

Variables	Statistics
Age—years	
Mean	56.4 ± 7.7
Median (IQR)	56 (50, 62)
Sex—no. (%)	
Female	252 (100)
Male	0
Weight—Kg	70.40 ± 11.31
Height—Cm	158.69 ± 6.81
BMI—Kg/m^2^	27.96 ± 4.29
Age of menstruation—years	13.3 ± 1.4
Age at first pregnancy—years	21.7 ± 5.4
Number of pregnancy—no. (%)	
1	18 (7.6)
2	56 (23.6)
3	62 (26.2)
4	42 (17.7)
5 or more	74 (24.9)
Menopause—no. (%)	
Yes	191 (76.1)
No	50 (19.9)
Not sure	10 (4)
Age at menopause—years	48.9 ± 4.5
Oral contraceptive use—no. (%)	
Yes	154 (61.1)
No	98 (38.9)
Hormone replacement therapy use—no. (%)	
Yes	5 (2.1)
No	235 (97.9)
Diabetes—no. (%)	
Yes	46 (18.3)
No	206 (81.7)
Hypertension—no. (%)	
Yes	71 (28.2)
No	181 (71.8)
Hypercholestrolemia—no. (%)	
Yes	73 (29)
No	179 (71)
Hypothyroidism—no. (%)	
Yes	39)15.5)
No	213 (84.5)
Hyperthyroidism—no. (%)	
Yes	6 (2.4)
No	246 (97.6)
Family history of breast cancer—no. (%)	
Yes	83 (32.9)
No	169 (67.1)
Cigarette use—no. (%)	
Yes	1 (0.4)
No	251 (99.6)
Waterpipe use—no. (%)	
Yes	24 (9.5)
No	228 (90.5)
Alcohol use—no. (%)	
Yes	1 (0.4)
No	251 (99.6)
Breast side involvement—no. (%)	
Right	121 (48)
Left	131 (52)

*IORT* intraoperative radiation therapy, *BMI* body mass index

*All plus-minus values are means and standard deviations unless stated otherwise

The mean tumor size among patients was 1.56 ± 0.55 cm. In histopathology, majority of patients had grade 1 (44.6%) and 2 (41.4%) BC. Moreover, with regard to grade of nucleus most patients had grade 1 (43.9%) and 2 (34.5%). In total, 71.7% of patients had in-situ components. The most common type of invasion was lymphovascular invasion which was seen among 14.5% of patients. Three patients in our report required additional surgery which included mastectomy. Moreover, two patients underwent re-excision of margins.

All of our patients were ER positive and majority of patients were PR positive (93.4%). In total, 14.6% of patients who underwent IORT had overexpression of HER2.

Regarding treatment specifics, 83.7% of patients received chemotherapy after breast conserving surgery. Most patients received hormone therapy (98.3%), from which patients mostly received letrozole alone (65.3%) followed by tamoxifen alone (24.2%) and the rest received either a combination of tamoxifen and laterazole (9.3%) or aromasins (1.3%).

In total, 11 patients (4.3%) with DCIS in our study received IORT. Overall 10 of these patients had DCIS components in the setting of invasive ductal carcinoma and one patient had DCIS component in the setting of tubular carcinoma. All these patients had free margins in histopathology examination and none experienced recurrence.

Mean (SD) and median (IQR) follow-up of patients were 36.3 ± 18.7 and 34.1 (23.1–51.2) months, respectively. Overall, 8 patients (3.1%) experienced recurrence in follow-up visits (disease-free-survival of 96.1%), among which four (1.5%) were local recurrence, two (0.8%) were regional recurrence and two patients (0.8%) had metastasis.

**Fig. 1:**
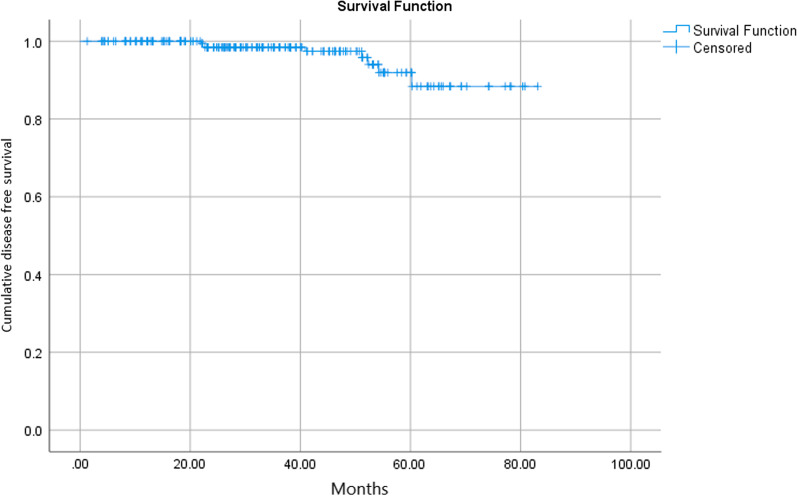
The figure shows the Kaplan–Meier plot for disease-free survival among patients who underwent IORT

Mean (SD) and median (IQR) time to recurrence were 32.1 ± 17.3 and 46 (22–53.7) months, respectively, among patients who had recurrence. Figure 1 shows Kaplan–Meier plot for disease-free survival.

Three patients in our series experienced pneumothorax which resolved after treatment. One patient died due to recurrence after 64 months of their primary diagnosis (Table 2).

**Table 2:** Clinical characteristics of patients who received IORT.*

Variables	Statistics
Type of breast surgery—no. (%)	
Quadrantectomy	249 (98.8)
Quadrantectomy + mastectomy	3 (1.2)
Type of axillary management—no. (%)	
SLNB	252 (100)
Pathology subtype—no. (%)	
Invasive ductal carcinoma	227 (90.1)
Invasive lobular carcinoma	6 (2.4)
Invasive tubular carcinoma	5 (2)
Mucinous	3 (1.2)
Papillary	3 (1.2)
Other	8 (3.2)
Tumor size—cm	1.56 ± 0.55
Tumor size—cm	
< 2	204 (87.2)
2–2.5	28 (12)
2.5–3	2 (0.9)
Tumor grade—no. (%)	
1	99 (44.6)
2	92 (41.4)
3	31 (14)
Grade of nucleus—no. (%)	
1	65 (43.9)
2	51 (34.5)
3	32 (21.7)
In-situ component—no. (%)	
Yes	157 (71.7)
No	60 (27.4)
Unknown	2 (0.9)
Tumor necrosis—no. (%)	
Yes	76 (38.6)
No	120 (60.9)
Unknown	1 (0.5)
Invasion—no. (%)	
Perineural	19 (8.6)
Lymphatic-vascular	32 (14.5)
All	10 (5)
None	159 (71.9)
Lymph node involvement	
No	231 (91.6)
Micrometastasis	21 (8.4)
Marginal involvement	
Negative	252 (100)
Esterogon receptor—no. (%)	
Positive	240 (100)
Negative	0
Progesterone receptor—no. (%)	
Positive	225 (93.4)
Negative	16 (6.6)
HER2 overexpression—no. (%)	
Positive	31 (14.6)
Negative	182 (85.4)
Chemotherapy before surgery—no. (%)	
Yes	0
No	252 (100)
Chemotherapy after surgery—no. (%)	
Yes	205 (83.7)
No	40 (16.3)
IORT type—no. (%)	
Radical	252 (100)
Hormone therapy—no. (%)	
Yes	236 (98.3)
No	4 (1.7)
Type of hormone therapy—no. (%)	
Tamoxifen	57 (24.2)
Laterazole	154 (65.3)
Tamoxifen + laterazole	22 (9.3)
Aromasins	3 (1.3)
Recurrence—no. (%)	
Yes	8 (3.1)
Local	4 (1.5)
Regional	2 (0.8)
Metastatic	2 (0.8)
No	246 (96.9)
Mortality—no. (%)	
Yes	1 (0.3)
No	251 (99.7)
Follow-up duration—months	
Mean	36.3 ± 18.7
Median (IQR)	34.1 (23.1, 51.2)

*SLNB* sentinel lymph node biopsy

*All plus-minus values are means and standard deviations unless stated otherwise

Detailed information on patients who experienced recurrence has been mentioned in Additional file 1.

## Discussion

We presented our experience with the use of IORT among patients with BC in our region using our own specific institutional criteria. We found that during a mean follow-up of 3 years, 3.1% of our patients developed recurrence, furthermore one patient died in our series.

IORT, which includes the delivery of intraoperative radiation to a specific tumor, has been used in many instances. Some of which include: head and neck cancers [14], colorectal cancers [15], soft tissue sarcomas [16], pediatric tumors [17], gynecological [18], genitourinary [19], prostate [20], pancreatic, gastroinestinal [21, 22] and BCs.

The ELIOT clinical trial study [4] compared standard EBRT and IORT. They used the following criteria for patient selection: age of 48–75 years and tumor size of ≤ 2.5 cm. In this study, authors did not consider hormone receptor status and pathology sub-type as criteria for patient selection. Comparing patients who received IORT to those who received external radiotherapy, they reported a 5 year loco-regional recurrence of 5.4% vs. 0.8% (p < 0.0001) and distant metastasis of 5.1% vs. 4.8% (p = 0.94, respectively. Moreover, they recorded a death rate of 3.2% among patients in the IORT arm. Majority of their patients in the IORT arm were between 50 and 70 years old (84%), had a tumor size of less than 1.5 cm (69%), had zero positive lymph nodes (74%), had grade 2 tumors (48%), were oestrogen (90%) and progesterone (76%) receptor positive. Most of their patients also received endocrine therapy alone (75%) compared to chemotherapy and combined endocrine and chemotherapy. In a recently updated report on this trial [11], authors evaluated the long-term outcomes of patients during a median follow-up of 12.4 years. They found a higher rate of recurrence among those in the ELIOT arm compared to those with WBI (11% vs. 2%, p < 0.001), although no difference was seen in overall survival between the two groups.

One of the largest studies that compared WBI and IORT, is the TARGIT-A study which was conducted in 11 countries [3]. In this study, they included women older than 45 years old with unifocal ductal carcinomas. Using low energy photon, they recorded a 3.3% rate of local recurrence and a 3.9% mortality rate among patients who received IORT during a median follow-up of 2 years and 5 months. In this report most of their cancers were grade 1 and 2 (85%), smaller than 2 cm (87%), ER positive (93%) and PR positive (82%). In a report with a longer follow-up of patients within the TARGIT-IORT study [10], during a median of 8.6 years, authors found no significant difference between the IORT group (n = 1140) and the EBRT group (n = 1158) regarding local recurrence-free survival (167 vs. 147, respectively; p = 0.28), mastectomy-free survival (170 vs. 175, respectively; p = 0.74) and mortality attributed to BC (65 vs. 57, respectively; p = 0.54). Overall, they concluded that TARGIT-IORT was non-inferior to EBRT during long term follow-up and concluded that TARGIT-IORT to be an effective alternative to EBRT for early-stage BC.

Following the two large clinical trial studies on IORT, other centers have further reported their institutional experiences with IORT for early stage BC [23–25]. Among which, chowdhry et al. [26] reported on 109 patient who received IORT from the Massachusetts General Hospital. They had a median follow-up of 29.9 months, during which 2 of their patients (1.8%) experienced local recurrence and one patient (0.9%) had regional recurrence. In this study they included only patients with T1N0 with smaller than 3 cm tumors that were estrogen positive. All their patients had a negative margin. Their median tumor size was 9.3 mm and majority of their patients had invasive ductal carcinoma (69.7%) followed by ductal carcinoma in-situ (27.5%). Three-year diseases-free-survival and overall survival were 97.2% (95% CI 88.9–99.3) and 96.0% (95% CI 84.9–99.0), respectively.

Patient selection for IORT is of vital importance and requires a multidisciplinary approach. Accordingly, we set-up our criteria based on the consensus of a joint committee of surgical oncologists, pathologists and radiation oncologists. We further compared our criteria with that of the two largest clinical trial studies on IORT (the ELIOT and the TARGIT-A) in Table 3.

**Table 3:** Comparison of inclusion criteria for IORT between our study and the ELIOT and TARGIT-A studies

Criteria	Our study	ELIOT	TARGIT-A
Age—yrs	≥ 45	48 ≤ age < 75	≥ 45
Histology sub-type	Invasive ductal carcinoma	Invasive carcinoma (unifocal)	Invasive carcinoma
	Lobular carcinoma (with MRI and radio-concologist confirmation)	Lobular carcinoma (with MRI confirmation)	No lobular
	DCIS (low and intermiediat grade, tumor size ≤ 2.5 sm and margin of 2–3 mm)	–	–
Tumor size	45–50 years old then 0–2 cm	≤ 2.5 cm	< 2 cm or < 3.5 (with N0-1 and M0 with cytology or histology confirmation)
	50–55 years old then < 2.5 cm	–	–
	> 55 years old then < 3 cm	–	–
Marginal status	Invasive tumor = negative margin	–	–
	DCIS ≥ 3 mm	–	–
Nodal status	Negative	–	–
	Or micro-metastasis	–	–
Hormone receptor status	ER +	–	–
Others	–	–	Previously diagnosed and treated contralateral breast cancer

*IORT* intraoperative radiation therapy, *DCIS* ductal carcinoma in-situ, *ER* estrogen receptor

In our series, we selected patients for IORT who were older than 45 years old, which was younger than the criteria of the American Society for radiation oncology [9]. This was mainly due to the fact that Iranians tend to show BC at a lower age compared to that of other regions in the world [27, 28]. When comparing our findings with that of other centers in the world, we had similar clinical outcomes with IORT. This shows that our modified criteria for patient selection results in good clinical outcomes. During our follow-up, we had one death due to distant metastasis. Our 96.9% 5-year disease free survival is similar to reports from other regions and centers of the world [23].

None of our 11 patients with DCIS experienced any recurrence during the mean follow-up of 36 months in our study. This shows that with careful patient selection those with DCIS will safely benefit from IORT.

Another interesting point was that almost one third of our patients had a family history of BC, this may be the result of the screening program of family members among individual with BC, which is applied in our center.

For the first time, we categorized patients according to age and tumor size and accordingly older patients with larger tumor sizes were considered appropriate candidates for IORT in our series. This specific classification was done as younger individuals usually demonstrate more aggressive tumor behavior [8, 29, 30]. Our experience showed that this method of patient-selection results in good clinical outcomes and can be implemented for IORT in other centers in the world. From another aspect, IORT provides a means for treatment of patients with early stage BC without the need for mastectomy and provides a much less costly method of delivering radiotherapy compared to external radiation.

This study was not without limitation and warrants further discussion. Although this was one of the largest single-center reports in literature and the largest in the Middle East, due to the low number of patients who had recurrence, conducting a separate analysis to determine the predictors of recurrence in this population was not feasible. As this is a study from one of the main referral centers in Iran and among few centers that have facilities for IORT, patients are referred from different regions of Iran, hence this report can be representative of the Iranian population. Considering that IORT is relatively new in the Middle East and guidelines on the use IORT are constantly being modified, certain modification to selection criteria of patients to receive IORT are expected according to characteristics of BC within each specific population. Facilities for IORT are still not widely available in Iran and in other centers in the Middle East, and our results may not be applicable in most centers due to lack of infrastructure. Our experience showed good outcomes at 36 months (median) follow-up, however longer follow-ups are still lacking due to the novelty of the procedure in our region and institution. In this study we merely reported on clinical outcomes and epidemiology of patients who received IORT in our institution, it would be interesting to cross compare these patients with those who had similar BC conditions and received WBI to evaluate percentage of local recurrence, as some studies have shown that these individuals will experience higher rates of local recurrence [31]. We had relatively high rates of chemotherapy among patients, a reason for this may be that according to the NCCN guidelines [32], all patients who have a hormone receptor positive and a HER2 overexpression negative status, with negative lymph nodes and a tumor size of > 5 mm should either have the oncotype test or should receive chemotherapy. Patients with early stage breast cancer and HER2 overexpression, must receive chemotherapy if their tumor size is > 5 mm. Unfortunately, the oncotype test is not available in our country and our oncologists discuss the benefits and side effects of chemotherapy with patients. Accordingly a high proportion of our patients are treated with chemotherapy.

## Conclusion

Our series shows our experiences with use of IORT in a region where facilities for IORT are limited using our own modified criteria for patient selection.

## Supplementary Information

**Additional file 1: Supplement Table**. Detailed information among patients with recurrence of primary cancer.

## Data Availability

Authors and institution may request the data from the study by directly contacting the corresponding author.

## Abbreviations

WBI: Whole breast irradiation
BC: Breast cancer
BCS: Breast conserving surgery
DCIS: Ductal carcinoma in-situ
ER: Estrogen receptor
PR: Progesterone receptors
HER2: Human epidermal growth factor 2
OCP: Contraceptive medication
SD: Standard deviations
IQR: Interquartile range
