# Supplementary material for: Intraoperative radiation therapy for early stage breast cancer
Source: BMC Surg. 2022 Jan 26;22:26. doi: 10.1186/s12893-021-01427-5 (PMC8793207; doi:10.1186/s12893-021-01427-5)
Supplement: Supplementary file 1 — Additional file 1: Supplement Table. Detailed information among patients with recurrence of primary cancer. [file 12893_2021_1427_MOESM1_ESM.docx]

| **Supplement Table.** Detailed information among patients with recurrence of primary cancer. | | | | | | | | |
| --- | --- | --- | --- | --- | --- | --- | --- | --- |
| **Variables** | **Patient number** | | | | | | | |
|  | 1 | 2 | 3 | 4 | 5 | 6 | 7 | 8 |
| Age - yrs | 65 | 47 | 64 | 56 | 64 | 46 | 60 | 67 |
| BMI - kg/m^2^ | 27.1 | 37.1 | 30.0 | 29.9 | 33.0 | 25.9 | 32.2 | 34.1 |
| Type of pathology | IDC | IDC | IDC | IDC | IDC | IDC | IDC | IDC |
| Tumor size - cm | 1.50 | 1 | 2 | 1.20 | - | 2 | 1.30 | 2 |
| Tumor grade | 2 | 2 | 3 | 2 | - | 3 | 2 | 3 |
| In-situ component in pathology | Yes | Yes | Yes | Yes | No | - | No | Yes |
| Tumor necrosis in pathology | Yes | Yes | Yes | Yes | No | No | No | - |
| Marginal involvement | No | No | No | No | No | No | No | No |
| Nodal involvement in SLNB | No | No | Yes (micrometastasis) | No | No | No | No | No |
| ER expression | Positive | Positive | - | Positive | Positive | Positive | Positive | Positive |
| PR expression | Positive | Positive | Negative | Positive | Positive | Positive | Positive | Positive |
| HER2 overexpression | Negative | Negative | Negative | Negative | Negative | Negative | Negative | Negative |
| Duration of follow-up - months | 64.2 | 63.7 | 58.1 | 66.5 | 51.2 | 69.2 | 58.1 | 32.1 |
| Type of recurrence | Metastasis | Local | Regional | Local | Local | Local | Metastasis | Regional |
| BMI: body mass index; SLNB: sentinal lymph node biopsy; ER: estrogen receptor; PR: progesterone receptor; HER2: human epidermal growth factor receptor | | | | | | | | |
